# Complementary and Alternative Therapies (CATs) in Nursing Education in Spanish Universities

**DOI:** 10.3390/nursrep14030169

**Published:** 2024-09-06

**Authors:** Belén Gutiérrez-Sánchez, José Gutiérrez-Gascón, Henrique da-Silva-Domingues, Rafael del-Pino-Casado

**Affiliations:** Department of Nursing, Faculty of Health Sciences, University of Jaén, 23071 Jaén, Spain; bgutierr@ujaen.es (B.G.-S.); hda@ujaen.es (H.d.-S.-D.); rdelpino@ujaen.es (R.d.-P.-C.)

**Keywords:** complementary therapies, alternative therapies, nursing education, university students

## Abstract

Background: The use of complementary therapies in the general population is increasing, so it is necessary to understand the training that health professionals receive in this type of therapy in their training plans, as they are often the primary source of information for patients. Our aim was to investigate Spanish universities that offer subjects on complementary therapies in their nursing degree programs. Methods: This study is an observational, descriptive, cross-sectional study. For this purpose, we used a document published on the website of the Ministry of Universities as the working document. Additionally, a literature search was conducted up to September 2023 in the PubMed database, along with reverse searches. Results: Out of a total of 62 universities, only 16 (29%) offer a subject related to this type of therapy, 27.5% (11) are public universities and 22.7% (5) are private universities, most of them being optional subjects. Conclusions: The training content on complementary care in nursing degree programs in Spanish universities is scarce, highlighting the potential benefit of expanding and promoting it, in line with the recommendations of the World Health Organization.

## 1. Introduction

Complementary and alternative therapies (CATs) can be defined as the use of practices and products of non-mainstream origin, including natural products, mind and body practices and other approaches apart from traditional Western medicine [1]. However, in practice, there is no consensus regarding the terminology and concepts used to refer to these therapies, resulting in various ways of naming and defining them [2]. In this regard, it is necessary to consider the meanings of the following terms [3]: (a) complementary or integrative: when a non-mainstream approach is used alongside conventional approaches and (b) alternative: when a non-mainstream approach is used in place of conventional approaches.

Health professionals understand that these therapies can serve as a complement to modern health methods, but in no case can they be an alternative to them.

The World Health Organization (WHO) in the first global strategy on traditional medicine that was developed during the period 2002–2005 used the following terms to refer to these therapies: traditional medicine (TM), where they included traditional Chinese medicine, Hindu ayurveda and various forms of indigenous medicine. When these same therapies were used in countries such as the United States or Europe, they were referred to as complementary and alternative medicine (MCA) [4].

Currently, the WHO [5] refers to this type of therapy as traditional medicine, which it defines as follows: “the sum of knowledge, skills and practices based on the theories, beliefs and experiences of different cultures, explainable or not, that are used in the maintenance of health and the prevention, diagnosis, the improvement or treatment of physical and mental illnesses”.

In recent decades, there has been a significant surge in the demand for and consumption of CATs, creating a landscape where professionals and patients alike are increasingly interested in these therapies [2].

Several studies have highlighted the widespread use of CAT among patients with various health conditions. For instance, usage rates stand at 89.7% among hemodialysis patients [6], 71.1% among rheumatology patients [7], 67.3% among patients with multiple sclerosis [8], 55.4% among individuals over 65 years during the COVID-19 pandemic [9], 53.6% among oncohematology patients (Sánchez 2014), 35% among adult patients with joint pain [10] and 23% among patients with inflammatory bowel disease [11]. However, there exists a gap in both the training of professionals in CAT [12] and in the available evidence regarding the efficacy and safety of many CATs due to limited research in this area [13].

An increasing number of studies are substantiating the efficacy of various CATs. For instance, a comprehensive study on music therapy in patients experiencing anxiety, stress and depression, encompassing findings from 10 systematic reviews conducted across the United States, Denmark, the Netherlands, Norway, Taiwan and South Korea, analyzed 186 studies involving 11,326 participants across palliative care, dementia and cancer contexts. The study revealed that three of the reviews indicated the potential beneficial effects of music therapy for depression and anxiety [14]. A systematic review with a meta-analysis investigating the impact of Tai Chi on fall prevention in frail elderly individuals concluded that Tai Chi yielded beneficial outcomes in preventing falls [15]. A report by the Agency for Health Technology Assessment of Andalusia on acupuncture treatment for chronic non-oncological pain revealed evidence of short-term pain reduction in low back pain, neck pain and shoulder pain compared to sham acupuncture or usual care [16]. In a systematic review with a meta-analysis by Hou, Wang [17], examining the impact of yoga on breast cancer-related fatigue, the findings indicated that yoga led to improvements in sleep quality, anxiety, depression and the overall quality of life.

In Spain, the Ministry of Health published a report on Natural Therapies, highlighting the absence of research and regulation in the field. The report compiled a total of 139 therapies, aiming to establish a potential foundation for future regulatory measures [18]. As a follow-up to this report, in 2018, the Plan for the Protection of Health against Pseudo-therapies was launched, with the objectives of evaluating knowledge and scientific evidence, promoting the dissemination and transparency of information and ensuring regulatory compliance [19].

In response to the growing demand and application of these therapies, several governments and official bodies are taking steps to improve the information and safety surrounding them. For instance, in the United States of America, the National Center for Complementary and Integrative Health (NCCIH) was established. In Europe, the European Parliament’s Committee on the Environment, Public Health and Consumer Protection conducted a study on ‘Non-Conventional Medicines’, examining the status of these therapies across member states. This report compiled information on legislation at both the EU and member state levels concerning the training of professionals to practice these therapies [20]. The World Health Organization (WHO) is presently engaged in the ‘WHO Strategy on Traditional Medicine 2014–2023′ project. This initiative seeks to provide valuable guidance to governments, health professionals and users considering traditional medicine. Central to its objectives are the establishment of regulations to govern these practices and the promotion of research to assess their safety and efficacy [21]. At the 76th World Health Assembly held in Geneva from 21 to 30 May 2023, a decision was made to extend this project until 2025; at the same time, the development of a new draft global strategy on traditional medicine covering the period 2025–2034 was approved [22]. The CAMbrella project [23] was initiated due to significant heterogeneity across European Union countries concerning legislation, research and the training of health professionals in complementary and alternative medicine (CAM). This project marked the first instance of funding being allocated for research in this field. CAMbrella addressed various topics including terminology, patient perspectives, research methodologies and legislative considerations.

In nursing, the use of CATs has been endorsed by various organizations for many years [12]. In a report by an expert committee on the Practice of Nursing, the WHO recommended that nurses receive training to guide clients in choosing between various complementary and traditional healthcare methods. Nursing education should equip nurses with an understanding of these methods, their compatibility with other treatments and their cultural acceptability [24]. Furthermore, the Association of Colleges of Nursing in the United States outlined core competencies for nurse education, which include competencies related to complementary therapies. These competencies entail developing an awareness of complementary modalities and their role in health promotion, as well as evaluating the integration of traditional and complementary healthcare practices [25].

The growing use of CAT has significant implications for nurses regarding patient care and safety [26]. Moreover, multiple studies indicated that patients are more inclined to discuss CAT with nurses rather than with general practitioners or other professionals [27]. However, the level of knowledge among nurses regarding CAT remains modest [28].

Despite previous endorsements, support and evidence, various studies to date have underscored the limited availability of complementary and alternative therapy (CAT) subjects in universities [13,29,30].

To our knowledge, there is currently no study evaluating the availability of complementary and alternative therapy (CAT) subjects in nursing degree programs in Spain. Integrating CAT training into the Bachelor’s Degree in Nursing curriculum would serve to highlight effective and safe CATs, enhancing nurses’ therapeutic capabilities and mitigating the use of pseudo-therapies by patients. Therefore, we undertook a study to assess the status and features of CAT subject offerings in Bachelor’s Degree in Nursing curricula across Spain for the academic year 2022–2023.

## 2. Materials and Methods

### 2.1. Design

This cross-sectional study was carried out following the recommendations of the guideline Strengthening the Reporting of Observational Studies in Epidemiology (STROBE) [31].

### 2.2. Population

All faculties or affiliated centers in Spain, whether public or private, that teach nursing degree courses were included in this study. No sampling was carried out, and all faculties and centers that teach this program were included.

In order to obtain data relating to Spanish universities where nursing degree courses are taught and to be able to consult their study plans to check whether they offer subjects related to CAT, a document published on the website of the Ministry of Universities of Spain (public data), which lists all the universities that offer this degree, was used to define the population of this study. No sampling process was used since the entire population was included in this study.

The total number of universities that offer the nursing degree was 62. After consulting the study plans for the nursing degree on the website of each of these academic institutions, 16 universities offer subjects related to CAT in their academic offerings. Some of these universities have several centers, with a total of 22 centers offering subjects with this content.

Therefore, the total population of this research is made up of 16 universities with 22 centers distributed in different cities.

### 2.3. Variables

The following variables were collected: the type of university (public or private); the type of center (institutional or affiliated); the presence of a CAT subject (yes/no), and if yes, the name of the subject; the nature of the subject (compulsory or optional); duration in credits according to the European Credit Transfer System (ECTS; one credit equivalent to between 25 and 30 h of work, theoretical and practical training) and the type of content (general, covering CAT broadly across populations, or specific, focusing on particular therapies or populations).

### 2.4. Procedure

Data collection was carried out between November 2022 and January 2023. To gather data from centers offering the Bachelor’s Degree in Nursing and to access their curricula to assess the availability of CAT subjects, admission data for the nursing degree in Spain [32] were used. Subsequently, the website of each university or center was visited to review the current curriculum and subjects offered for the academic year 2022–2023.

Data were extracted independently by two of the authors, and discrepancies were resolved by a third author.

### 2.5. Data Analysis

The data obtained from the different databases consulted were grouped in the Microsoft Excel 2019 program for subsequent analysis. A descriptive analysis was carried out from the grouped relative measures. For quantitative variables, the analysis was carried out by calculating measures of central tendency such as the mean ($\bar{x}$) and measures of dispersion such as the standard deviation (SD), while the qualitative variables were analyzed using frequencies and percentages (%).

### 2.6. Ethical Aspects

The data for this study were obtained from the websites of Spanish universities that are publicly accessible. No personal data were included, and no interventions were conducted for individuals, so following the guidelines of the Ethics Committee of the University of Jaén, it was not necessary to request a report from this committee [33].

## 3. Results

A total of 62 Spanish universities offer the Bachelor’s Degree in Nursing, comprising 40 public institutions (64.5%) and 22 private ones (35.5%). Additionally, there are 55 centers where the degree is offered, with 29 being affiliated centers and 26 being institutional ones. Thus, there are a total of 117 centers across Spain offering the Bachelor’s Degree in Nursing. There were no missing data in this study.

In Table 1, out of 62 universities, 16 (29.0%) offer CAT subjects. Among these, eleven are public (68.8%), and five are private (31.3%). This indicates that 27.5% of public universities and 22.7% of private universities offer such subjects. In terms of centers (Table 1), 22 out of 117 (18.8%) offer CAT subjects.

In terms of the type of subject offered, CAT subjects are compulsory in two publicly owned centers, optional in nineteen and one center offers it as a free-choice subject.

Regarding the names of the subjects, 16 of them are described as “complementary” (with 12 specifically naming therapies). Additionally, the term “alternative” appears in four subjects, with one of them also including “complementary” in its description.

The duration of the subjects is measured in ECTS credits, which is the system used by the universities of the European Higher Education Area in which an estimate is made of the time needed by students to complete all the activities that allow them to obtain the learning results: classes, seminars, projects, practical work and freelance work. One ECTS credit is equivalent to between 25 and 30 h of work [34]. Credits range between three and six credits for both public and private centers ($\bar{x}$ = 3.9, SD = 1.4). For public centers specifically, the mean and standard deviation are 3.9 and 1.4, while for private centers, they are 3.8 and 1.5, respectively.

Out of the 22 subjects offered, 19 incorporate general content. Two subjects focus on specific therapies (mindfulness and lifestyles and thermalism), while one is tailored for individuals with cancer.

## 4. Discussion

To our knowledge, our study represents the first analysis of the provision of CAT subjects in the nursing degree in Spain. Our findings reveal that 29% of Spanish universities and 18.8% of centers offering nursing studies include CAT subjects in their nursing degree programs. The majority of these subjects are optional and have a duration of three credits. Notably, we did not observe significant differences between public and private institutions.

Our findings are consistent with those of similar studies conducted in various countries. For example, in a study by Fenton et al. [35] on the integration of complementary and alternative therapies into nursing school curricula in the United States, 125 out of 585 schools (21%) responded to an online questionnaire. While 97% (121) of the schools offered or planned to offer courses on CAT, only 19 (15%) had compulsory CAT courses, and 46 (36.8%) offered optional subjects. It is worth noting the potential selection bias in the study, as those most motivated by the subject may have been more likely to respond, suggesting that the actual prevalence of CAT subjects across all schools may have been higher than observed in the sample.

Studies carried out by Al-Rukman et al. [29], Valarezo-García et al. [30,36] and Salles et al. [37] analyze the situation of CAT subjects in different countries: Saudi Arabia, Ecuador, Peru and Brazil. If we compare their results with ours, we observe how Al-Rukman et al. [29] analyze the situation within faculties of health sciences between 1971 and 2011, collecting data from 15 faculties of nursing out of a total of 16. The study revealed that during the periods 1991–2000 and 2001–2011, 33% and 66% of the 15 nursing faculties, respectively, offered CAT subjects. They obtained a higher percentage of offering this subject with respect to Spain.

The number of universities studied in Ecuador and Peru was very similar to ours, 59 in Ecuador, 61 in Peru and 62 in Spain. Of the 59 universities studied by Valarezo-García et al. [33], only 21 offered nursing (17 public and 4 private), and of these, only 2 public universities offered courses related to CAT, for 9.5% of the total. This figure is much lower than in Spain.

However, in Peru, 61 universities that offered nursing courses were analyzed, of which 26 (42.6%, including 7 public and 19 private institutions) offered CAT subjects or modules. This represented 31.8% of public universities and 48.7% of private universities. If we compare this with our study, we observe very similar total percentages of supply; however, the percentage of offering these courses in Spain is higher in public universities, but in Peru, the opposite is true.

Salles et al. [37], on the other hand, analyzed nursing schools in Brazil and found that only 26.1% offered CAT subjects, of which only 26% were compulsory, unlike the results obtained in our study, which reveal that in Spain, most of the courses offered are optional.

We observed that in previous studies, the provision of subjects related to CAT was less than 50% of the universities or centers offering the nursing degree, failing to reach a quarter of these in Spain.

We believe that the recommendations of official organizations, coupled with the growing interest in these therapies and the demonstrated effectiveness of several, can significantly enrich the nursing therapeutic arsenal. Moreover, the imperative to distinguish between effective and ineffective therapies, in light of the nursing profession’s educational and counseling roles as discussed in the Introduction, warrants the widespread inclusion of these therapies in undergraduate nursing studies.

Conversely, the inadequate provision of CAT subjects fosters skepticism. This skepticism not only discourages the pursuit of further training but also has adverse effects on the development and integration of these interventions into practice [13,38].

Hence, we strongly advocate for the promotion of CAT teaching in nursing studies in Spain, urging political decision-makers to recognize its importance.

In terms of limitations, it is important to emphasize that the findings of our study are confined to the specific context of Spain. Although we observed consistency with studies conducted in other countries, the extrapolation of our results to international contexts should be approached with caution, considering cultural and organizational variations among healthcare systems and professional practices.

## 5. Conclusions

The provision of CAT training in Spain’s Bachelor’s Degree in Nursing programs is exceedingly limited. Therefore, it is imperative for political decision-makers to expand and promote the academic offerings of CAT in nursing programs across Spain.

## Figures and Tables

**Table 1:** Offer of complementary and alternative therapy subjects by universities and centers. Spain, academic year 2022–2023.

		Total	Offer		
			n	% of Total Universities/Centers	% of Universities Offering
**Universities**		62	16	25.8	
	Public	40	11	27.5	68.8
	Private	22	5	22.7	31.3
**Centers**		117	22	18.8	

## Data Availability

All data are available from the authors upon reasonable request.

## Public Involvement Statement

There was no public involvement in any aspect of this research.
